# *“The talking bit of medicine, that’s the most important bit”*: doctors and Aboriginal interpreters collaborate to transform culturally competent hospital care

**DOI:** 10.1186/s12939-021-01507-1

**Published:** 2021-07-23

**Authors:** Vicki Kerrigan, Stuart Yiwarr McGrath, Sandawana William Majoni, Michelle Walker, Mandy Ahmat, Bilawara Lee, Alan Cass, Marita Hefler, Anna P. Ralph

**Affiliations:** 1 Menzies School of Health Research, Charles Darwin University, PO Box 41096, Casuarina, Darwin, NT 0811 Australia; 2 Royal Darwin Hospital, Darwin, NT 0811 Australia; 3 Flinders University, Northern Territory Medical Program, Darwin, NT 0815 Australia; 4 Aboriginal Interpreter Service, Northern Territory Government, GPO Box 4396, Darwin, NT 0801 Australia; 5 Charles Darwin University, PO Box 41096, Casuarina, Darwin, NT 0811 Australia

**Keywords:** Cultural safety, Health, Racism, Communication, Aboriginal, Interpreters, Healthcare communication

## Abstract

**Background:**

In hospitals globally, patient centred communication is difficult to practice, and interpreters are underused. Low uptake of interpreters is commonly attributed to limited interpreter availability, time constraints and that interpreter-medicated communication in healthcare is an aberration. In Australia’s Northern Territory at Royal Darwin Hospital, it is estimated around 50% of Aboriginal patients would benefit from an interpreter, yet approximately 17% get access. Recognising this contributes to a culturally unsafe system, Royal Darwin Hospital and the NT Aboriginal Interpreter Service embedded interpreters in a renal team during medical ward rounds for 4 weeks in 2019. This paper explores the attitudinal and behavioural changes that occurred amongst non-Indigenous doctors and Aboriginal language interpreters during the pilot.

**Methods:**

This pilot was part of a larger Participatory Action Research study examining strategies to achieve culturally safe communication at Royal Darwin Hospital. Two Yolŋu and two Tiwi language interpreters were embedded in a team of renal doctors. Data sources included interviews with doctors, interpreters, and an interpreter trainer; reflective journals by doctors; and researcher field notes. Inductive thematic analysis, guided by critical theory, was conducted.

**Results:**

Before the pilot, frustrated doctors unable to communicate effectively with Aboriginal language speaking patients acknowledged their personal limitations and criticised hospital systems that prioritized perceived efficiency over interpreter access. During the pilot, knowledge of Aboriginal cultures improved and doctors adapted their work routines including lengthening the duration of bed side consults. Furthermore, attitudes towards culturally safe communication in the hospital changed: doctors recognised the limitations of clinically focussed communication and began prioritising patient needs and interpreters who previously felt unwelcome within the hospital reported feeling valued as skilled professionals. Despite these benefits, resistance to interpreter use remained amongst some members of the multi-disciplinary team.

**Conclusions:**

Embedding Aboriginal interpreters in a hospital renal team which services predominantly Aboriginal peoples resulted in the delivery of culturally competent care. By working with interpreters, non-Indigenous doctors were prompted to reflect on their attitudes which deepened their critical consciousness resulting in behaviour change. Scale up of learnings from this pilot to broader implementation in the health service is the current focus of ongoing implementation research.

## Introduction

Effective communication between doctors and patients is a determinant of patient outcomes, and vital for the delivery of culturally safe care [1–5]. In Australian hospitals, language discordance for patients who speak English as a second language is common. Interpreter mediated healthcare has been shown to improve clinical outcomes and contribute to higher patient satisfaction [5–8], however professional interpreters in healthcare are underused globally [6, 8–10].

This is particularly concerning in the Northern Territory (NT) of Australia where Aboriginal peoples experience an extremely high burden of disease and 60% speak an Aboriginal language as their first language [11]. The NT is the heartland of Aboriginal languages in Australia. Of the 14 languages identified nationally as “relatively strong”, 12 are in the NT [12, 13]. In the Top End of the NT, between 60 and 90% of patients presenting to hospitals and clinics are Aboriginal. Life expectancy of Aboriginal peoples in the NT is the lowest in Australia (66 years for males and 69 years for females) [14] and the prevalence of rheumatic heart, cardiovascular, lung and end-stage kidney disease and psychological distress are disproportionately high [15]. Ineffective health communication in the NT has resulted in death [16, 17]; absence of informed consent, unnecessary elongated hospital stays; discharge against medical advice and distrust of healthcare providers [5, 18–20]. Research has also found one of the most common ways patients experience racism is through poor communication [16, 21–23]. Intercultural communication challenges are also a stressor for healthcare providers who can “experience a sense of hopelessness” [24] when language discordance occurs. However when providers work effectively with interpreters the quality of medical care improves [6]; this includes a reduction in unnecessary diagnostic tests [25] and duration of hospitalisation stays [26]. Interpreter-mediated communication between patient and provider also means miscommunication is “much less likely” [6] which results in reducing the prospect of medical errors attributable to communication issues.

At the NT’s largest hospital, Royal Darwin Hospital (RDH), it is estimated around 50% of patients would benefit from an interpreter, yet only approximately 17% get access [27], despite face to face and telephone interpreting services being available. The underuse of interpreters in healthcare is commonly blamed on limited interpreter availability, healthcare provider time constraints and the perception that use of interpreters in healthcare is an anomaly [6, 7]. However, even when interpreters are readily available, doctors tend to communicate without an interpreter, utilising the physician centred style of communication which focuses on gathering clinical data and limits opportunities for shared decision making and person-centred care [7]. To overcome these constraints and attempt to normalise the presence of Aboriginal language interpreters, we conducted a pilot study which embedded Yolngu Matha and Tiwi interpreters in a team of renal doctors at RDH. The Yolngu and Tiwi patient experience of the pilot study has been previously reported [5]. We found by embedding Aboriginal language interpreters in the renal team, the power dynamics between doctors and Aboriginal clients changed. With consistent access to interpreter mediated communication patients determined the care they received was culturally safe. Before the pilot, with limited or no interpreter access, patients described feeling “stuck” and disempowered when forced to communicate in English. After receiving access to trusted interpreters who shared patients’ worldviews, patients said they felt empowered and “satisfied” with their care [5].

Jennings et al. [4] argued by changing how healthcare providers speak with Aboriginal clients, “we can alter the power dynamics and cultural safety of health consultations”. Cultural safety places the onus for change on providers and institutions to reflect on their own culture and acknowledge the “biases, attitudes, assumptions, stereotypes, prejudices, structures and characteristics” which impede the delivery of equitable care [28]. To deliver culturally safe care, healthcare providers and the institutions in which they work, also need to be culturally competent. Culturally competency requires an ongoing commitment to respect and respond to cultural diversity [29] thereby creating the opportunity to deliver culturally safe care. Both cultural competency and cultural safety avoids problematizing Aboriginal peoples by focusing on creating individual and systemic change through critical reflection [3, 4, 28, 30, 31]. The aim of this paper is to document the process of self-reflection, and subsequent changes, undertaken by RDH doctors and Aboriginal language interpreters who worked side by side during the 4-week pilot study.

## Methods

### Study design

This pilot study which embedded Aboriginal language interpreters in a RDH renal medical team is part of a larger Participatory Action Research (PAR) [32–34] project in which participants and researchers collaborated to address barriers to culturally safe communication at RDH. During data collection for the larger PAR project [27, 35–37] co-author SWM, and other doctors, expressed frustration regarding limited access to Aboriginal language interpreters at RDH and imagined the benefits of interpreters embedded in the multi-disciplinary team (MDT). Discussions with the NT Aboriginal Interpreter Service (AIS), RDH and researchers followed, and all agreed to pilot and evaluate the idea. The projects’ conceptual framework was influenced by cultural safety [3] and critical race theory [38] which both draw on Habermas’ approach to critical theory [39, 40]. Of particular relevance is Habermas position that communication can be used to address power structures which create and maintain inequities [39, 40].

### Researcher reflexivity

The lead author VK is an English speaking Australian born White researcher [41]. The second author SYM is a Gumatj man from the Yolŋu nation in north east Arnhem Land in the NT. SYM is bilingual: he speaks Djambarrpuyŋu, a dialect of Yolŋu Matha and English. Reflecting on the propensity of White researchers to perpetuate a “politic of domination” [42], the PAR project was designed collaboratively with Aboriginal researchers, interpreters, community leaders and healthcare providers who shared a commitment to social justice. As per PAR, this approach ensured the research addressed local priorities and findings could be translated into practice [32, 34].

### Study context

RDH is a 360-bed facility managed by the NT government’s Top End Health Service (TEHS), on Larrakia country in the capital of the NT. The pilot study was conducted at the inpatient renal unit where 84% of patients identified as Aboriginal [5]. Whilst English is the operational language of RDH, it is not the language most spoken amongst renal patients: 78% of Aboriginal renal patients spoke one or more of the 15 Aboriginal languages identified [5]. The most spoken languages were Yolŋu Matha and Tiwi, followed by Kunwinkju, Anindilyakwa and Kriol [5]. At the time of the pilot, Aboriginal language interpreters for RDH were provided by the NT AIS via a bookings system. The NT AIS is funded by the NT government which provides qualified interpreters to both government and non-government agencies including health, legal and community service organisations. Depending on interpreter availability, the NT AIS also provided one interpreter to RDH every weekday morning for 4 hours. The study was divided into two 2-week periods to align with specialist SWM’s roster. The NT AIS initially agreed to supply one Yolŋu Matha interpreter to work with the renal team led by SWM during morning ward rounds when important clinical decisions were made. The decision to focus on Yolŋu Matha was based on both the predominance of Yolŋu languages and pragmatism: the NT AIS employed experienced Yolŋu Matha interpreters and researcher SYM spoke the dialect Djambarrpuyŋu as his first language. After further assessing the language needs of the patient cohort, Tiwi interpreters were also employed. Two of the strongest Aboriginal languages are Tiwi (> 2000 speakers) and Djambarrpuyŋu, a dialect of Yolŋu Matha (> 4000 speakers). Yolŋu Matha is a group of mutually comprehensible languages of the Yolŋu people from North-East NT [12].

### Participants

Consistent with PAR [32, 43], doctors and NT AIS staff were purposively sampled based on their work roster, anticipated capacity to contribute to “the development of knowledge” [44] and commitment to the aims of the pilot. All participants provided written consent to participate. As per PAR, some doctors and NT AIS staff had roles as both co-researchers and participants. Doctors were only eligible to participate if they had worked in the Top End for more than 12 months and planned to remain in the region for 12 months or more. This selection criteria had a dual purpose: it meant doctors could reflect on their practice pre-pilot, report changes and consolidate learnings and be a potential catalyst for systemic change. We acknowledge doctors have the capacity to be transformational leaders in their teams and amongst hospital executive and policy makers [45].

### Data collection

Data sources included semi-structured interviews conducted by VK in English, field notes which documented patient-interpreter-provider interactions and doctors journals. Pre pilot, lengthy interviews provided an opportunity for doctors and NT AIS staff to reflect on their own behaviour and the systems they work in. During the pilot, to gain a deeper understanding of attitudes and behaviour, VK and SYM shadowed doctors during ward rounds, staff meetings and breaks and doctors wrote journal entries for each day they worked on the pilot.

### Data analysis

A critical theory [40] lens which examined power relations and explored multiple realities considering social, political and cultural context shaped analysis. Interviews were transcribed verbatim. Inductive narrative analysis [46] of interview transcripts, doctors’ journals, and researcher field notes was conducted using NVivo12. First round analysis entailed coding transcripts, journals and field notes separately. The second round of analysis merged the separate codes to identify turning points and “transformative emotional growth experiences” [47] for both doctors and interpreters. Co-authors then iteratively refined findings guided by the literature and drawing on both personal and professional experiences. For reporting purposes, participants were given a choice of using a pseudonym or their own name: co-researchers and participants SWM (Dr William) and the NT AIS trainer MA (Mandy) are identified in the paper according to their wishes.

### Ethical considerations

Regarding terminology, the language group, or associated nation, of Aboriginal participants will be used. Otherwise, the term Aboriginal, which refers to the original occupants of mainland Australia, will be used. The term White is capitalised in line with Whiteness studies. White refers to a social category which describes individuals who participate in “racialized societal structure that positions them as “White“ and accordingly grants them the privileges associated with the dominant Australian culture.” [41] Approval to conduct the study was provided by the Northern Territory Department of Health and Menzies School of Health Research Ethics Committee.

## Results

The pilot occurred in the RDH renal department over two periods in 2019: 14th to 27th of August (10 days) and 25th November to 3rd December (7 days). Period 2 was shorter due to NT AIS resourcing issues. Twelve interviews were conducted, comprising baseline and follow up interviews with three doctors, the two Yolŋu Matha interpreters and an interpreter trainer. The Tiwi interpreters consented to be observed only. The renal team and interpreters were shadowed by VK, and SYM when appropriate, during medical ward rounds which occurred between the hours of 8 am to 2 pm for a total of 29 h across 7 non-consecutive days. Twenty-one patient-interpreter-provider interactions were observed (15 Yolŋu Matha, 5 Tiwi, 1 Ngan’gikurunggurr).

Three male doctors from the RDH renal team participated. Dr. William was a specialist nephrologist, who had worked in the Top End for a decade. He was trained in Zimbabwe and the United Kingdom. From the Shona tribe in Zimbabwe, he spoke two African languages (Shona and Ndebele) and English. Dr. Sean was a medical registrar who had worked in Darwin for 12 months. He completed his medical training in Northern Ireland where he was born. He grew up speaking Gaelic and said he viewed the world through an *“Irish Catholic lens”.* Dr. Jack was an Australian trained medical registrar who had worked in the Top End for 4 years. He described his background as Anglo Celtic conservative, Christian and privileged. He was a monolingual English speaker. Dr. William participated in period 1 and 2. Due to the nature of their work roster, Dr. Sean participated in period 1 and Dr. Jack in period 2. Observations of the multi-disciplinary renal team (other doctors, nurses, allied health) were documented by VK and will be presented anonymously.

Two Yolŋu Matha interpreters, two Tiwi interpreters and an interpreter trainer participated. All interpreters were employed by the NT AIS on a casual basis. Period 1 Yolŋu interpreter Carly worked previously as an Aboriginal Health Practitioner and subsequently as an interpreter for over 12 months. Period 2 Yolŋu interpreter Joanna recommenced work at the NT AIS 1 week before period 2 started. Joanna had a long professional history, including as a nationally accredited interpreter, and more recently holding managerial positions in mainstream institutions. The period 1 Tiwi interpreter was employed 2 days before starting work on the pilot. The period 2 Tiwi interpreter had been employed on a casual basis by the NT AIS for more than 12 months. During the pilot, interpreters were supported by NT AIS trainer Mandy. Mandy was born in Darwin; she has Aboriginal and Torres Strait Islander heritage with connections to the Nyikina and Ngalakgan peoples and Badu Island. Mandy’s primary role was to support interpreters, although as her knowledge of the hospital developed she also supported doctors and patients by booking extra Aboriginal interpreters as required. This will be discussed in more detail below. Participant details are also presented in Table 1.

**Table 1:** Interpreter ward round pilot participants

	Period 1	Data collected	Period 2	Data collected
**AIS staff**				
**Yolŋu Interpreter**	Carly: AHP; interpreter at NT AIS for > 12 months	Interviews; observation	Joanna: experienced interpreter; former manager at a government department; interpreter at NT AIS for 1 week	Interviews; observation
**Tiwi Interpreter**	Name withheld: interpreter at NT AIS for 2 days	observation	Name withheld: interpreter at NT AIS for > 12 months	observation
**Interpreter trainer**	Mandy: NT AIS trainer for four years. Aboriginal and Torres Strait Islander heritage: connections to the Nyikina and Ngalakgan peoples and Badu Island.	Interviews; observation	Mandy also participated in period 2	
**Doctors**				
**Specialist**	William: born Zimbabwe; multilingual; Top End > 10 yrs	Interviews; observation, journal	William also participated in period 2	
**Registrars**	Sean: born Northern Ireland; multilingual; Top End > 2 yrs	Interviews; observation, journal	Jack: born Australia; monolingual; Top End > 4 yrs	Interviews; observation, journal
**Members of the Multi-disciplinary team (MDT)**	Unnamed doctors, nurses, allied health professionals	Observation	Unnamed doctors, nurses, allied health professionals	Observation
**Patients (reported in Kerrigan et al** **[**5**])**				
**Aboriginal**	51 Aboriginal patients; 40 Aboriginal language speakers.	Observation	39 Aboriginal patients; 30 Aboriginal language speakers.	Observation
**Non-Aboriginal**	4 non-Indigenous patients. 4 patients unknown heritage.		5 non-Indigenous patients. 4 patients unknown heritage.	

To document any potential transformation amongst hospital-based healthcare providers, findings will be presented as a timeline: pre pilot, the pilot and post-pilot

### Pre-pilot: individual and institutional issues

Before the pilot, doctors and interpreters reflected on hospital culture regarding communication and working with interpreters at RDH. Participants discussed attitudes and systems which bolster the idea that culturally safe communication is not a key component of running the hospital and also explored the barriers to consistently working with interpreters at RDH.

#### Hospital culture

Doctors reported that, patient centred communication is not prioritised due to the way hospital processes are implemented. Dr. Jack journaled about the dominant attitude which concerned him: “*we are here to ‘do’ medicine, not the soft stuff”.* As communication is not prioritised, the responsibility for effective communication is left with the patient. A theme which consistently arose in interviews, journals and observations was that health providers justify communicating without an interpreter because a) they have determined the patient speaks “good English” and b) the patient did not request an interpreter. When communication goes awry, the patient is blamed and often labelled “non-compliant”. Dr. William admitted when he started working in the NT, his preconceived ideas impacted his approach to patients:

*“I had this view, which is actually a very skewed view, which a lot of health care professionals bring here with them from down south or from overseas, that Aboriginal people are non-compliant; they don’t listen”* – Dr William

The Top End has a high level of transient health staff. According to Dr. Jack, some come to Darwin for *“a short time or a good time*” to undertake training and others were “*tired”* long term staff, resistant to change. Many overseas trained health providers arrive in the Top End unfamiliar with Aboriginal cultures and the impact of colonisation. After starting work at RDH Dr. Sean, who migrated from Northern Ireland, said he was *“absolutely distraught at seeing people my age or younger on dialysis or dead or incredibly sick”.* As immigrants to Australia, both Dr. Sean and Dr. William acknowledged their own cultural background influenced their decision making. Dr. William saw similarities between the culture of Zimbabwe and Aboriginal cultures: respect for Elders, caring for the environment, concept of time and the impact of westernisation. But he said not all healthcare providers have the capacity to reflect and empathise with the patient. He provided the following example regarding speaking English:

*“I put myself in the patient’s position as I was when I was learning English and imagining a doctor speaking to me in English at that stage. I wouldn’t have understood anything they were saying … .and a lot of our doctors here are immigrants or they’ve come to Australia, so we should understand better.*” - Dr William

The hospital often operates above capacity, resulting in pressure to process, treat and discharge patients quickly. Dr. Jack understood the benefits of interpreter-mediated communication but explained he doesn’t use interpreters because the hospital’s priority is “*staffing and budgets and chaos and patient numbers in bed block”*. Dr. Jack said it’s “*like the patients aren’t even there”.* Exemplifying the pressure frontline care providers contend with, during the pilot, Dr. William received a page from hospital executive: “experiencing extreme bed pressure” (VK field notes 26/11/19). The pressure was on to discharge existing patients to vacate beds. Accelerated discharge and associated poor communication can lead to subsequent unplanned readmission. This cycle of discharge and readmission due to poor communication contributed to negative perceptions of Aboriginal patients who were labelled “*frequent flyers”.* Dr. Jack said that patients who are readmitted frequently are perceived as a *“chore*” and “*an inconvenience in your day”.* Doctors explained that stereotyping of patients results in “othering” of Aboriginal peoples in the hospital, as in the wider community:

*“there’s a lot of talk of ‘them’ and ‘they’ … and all the stereotypes associated with that, and rarely do the two mix except in our eyes in healthcare and in the courts … . particularly in a place like Darwin, it’s pretty much segregation still”.* - Dr Jack

Social segregation means interactions between non-Aboriginal and Aboriginal peoples are commonly limited to the hospital and the justice system which resulted in biased views, as lamented by Dr. Jack describing the system he works within:

*“I guess we see them – ‘them’ again, here I go again but – patients as perpetrators or they’re deviant, or they’re victims, really. I guess in other settings, in more community-based settings, you see more patients and you can see a broader spectrum of community lives.”* – Dr Jack

#### Difficulty accessing interpreters

Attitudes contributed to interpreter uptake and availability. Pre-pilot, accessing interpreters in the hospital was described by Dr. William as “*extremely difficult”.* Three main reasons were identified to explain this. Firstly, there is a small pool of Aboriginal interpreters in the NT. Having worked in other Australian hospitals which serviced migrant non-English speaking populations, Dr. Jack said accessing interpreters via a telephone hotline was easy compared to accessing Aboriginal interpreters. Dr. Sean shared his experience of trying to book a Burarra interpreter over 10 days for a chronically ill patient with cancer. Unable to book an interpreter and facing pressure from the hospital to discharge the patient, the team’s specialist decided to deliver the diagnosis in English. The complex conversation required an explanation of the patients swollen stomach. Dr. Sean said *“because of the swollen belly and the actions that were being demonstrated”* the patient thought she was pregnant. Doctors discovered this through a conversation with the patient’s family. An interpreter was subsequently able to be accessed to explain the patient was not pregnant but in fact had cancer.

Secondly, there is a perception amongst hospital staff that using Aboriginal interpreters is unnecessary, disrupts workflow and is a waste of scarce resources. The disposition of hospital staff was noted by interpreters who reported feeling unwelcome. Interpreter Joanna described doctors as “*intimidating”* and “*just like police*”. Many interpreters chose not to take hospital jobs because they had a bad experience or had heard from colleagues the hospital was an unpleasant place to work:

*“most of the interpreters don’t like coming back here because I think they find the staff rude or something, that they don’t speak to them”.* - Carly, Yolŋu Matha interpreter

Thirdly, Aboriginal interpreters themselves deal with a large burden of illness. One interpreter was treated in the Emergency Department twice during a 5-day period around work commitments. Another interpreter’s grandmother was an RDH inpatient and every day after her shift, she cared for her grandmother:

*“working with the pilot was hard for me because my grandmother was in hospital and I just kept getting calls from her because my mum was away at* [an East Arnhem community] *for a funeral. So my brother and I had to rotate around for her but my brother was also sick so it was just me.”* – Carly, Yolŋu Matha interpreter

Funerals are prominent in the lives of Aboriginal interpreters. Mandy explained Period 2 was delayed because a Yolŋu leader died which meant six Yolŋu interpreters were *“all out on sorry business”.* “Sorry business” broadly refers to funerals and associated cultural practices.

### The pilot: changing systems, developing knowledge and challenging attitudes

To integrate interpreters into medical teams during ward rounds, doctors adapted their work routines which resulted in improved knowledge of Aboriginal cultures, improved interpreter health literacy and an attitudinal shift amongst both doctors and interpreters.

#### Changing the work routine

Four areas of change were noticed: 1) doctors adapted their training schedule, 2) patient language needs were included in clinical conversations, 3) the duration of bedside consults lengthened and 4) the use of Aboriginal language interpreters, beyond Tiwi and Yolŋu Matha, increased.

Firstly, to ensure doctors had some knowledge of how best to work with Aboriginal interpreters, and knowledge of NT Aboriginal languages, the NT AIS offered a one-hour training session before both pilot periods to the renal doctors. Team leader, Dr. William said he had to *“squeeze in”* the working with interpreter training sessions amongst the heavy clinical training load. However, after attending sessions in period 1 and 2, he determined the training was invaluable and should be mandated. Dr. Sean journaled (19/8/19) the training reminded doctors to avoid medical jargon, use plain English and to communicate concisely: “*There are many who recite an essay before allowing the interpreter to speak”*.

Secondly, patient language needs were discussed during pre-ward round meetings when clinical plans were developed. This was an immediate change which was observed on Day 1 of period 1. Language requirements were known because the day before the pilot began, following researchers request, Dr. Sean asked each patient what language they spoke at home. With interpreters and researchers present in the pre-ward round meeting, doctors reviewed treatment plans and for the first time each patient’s language was discussed. Researcher VK observed the following. The registrar Dr. Sean briefed the team: he introduced each patient by name, language spoken and then discussed their condition. The first patient was from Borroloola, the specialist Dr. William said: *“Do you know I cover Borroloola, but I don’t know what language they speak.”* Next was a patient from Groote Eylandt who spoke Anindilyakwa. Dr. William said: “*I didn’t know there was a language like that.”* The language needs of a Tiwi patient were discussed, and Dr. William revealed he was unaware there were two Tiwi languages: modern and traditional. He asked NT AIS trainer Mandy to explain the difference between them. Dr. William appeared to be exposing his lack of knowledge as a learning opportunity in front of his junior staff (VK field notes 14/8/19). Over 10 days, this new pattern of discussing patients was standardised. Dr. Sean said this led to a shift in care as patients were considered in terms of *“Who they are, rather than what they are”.*

Another obvious consequence of embedded interpreters was the length of bedside consults with Yolŋu and Tiwi patients increased from 5 to 10 min to 40 min to 1 h. Drs William, Sean and Jack deemed this necessary to make up for years of miscommunication. Dr. Sean said*: “things take longer when you’re actually speaking to your patients”.* Dr. Jack said spending time communicating in the patient’s first language resulted in better time management overall: *“you spend less time chasing your tail, miscommunicating about something over and over again”.* Ward rounds which previously finished before midday were now continuing until mid-afternoon, meaning paperwork was not completed in a timely manner. Dr. Sean said a lengthy ward round should not be blamed on interpreters but on the doctors, who were learning how to work in a culturally safe system. However, the lengthy interpreter-mediated consults caused some disharmony amongst the renal team who noticed other language speakers were neglected. This caused an argument amongst doctors concerned that Tiwi and Yolŋu patients were receiving preferential treatment:

*“it’s frustrating that patients who don’t speak Tiwi or Yolŋu Matha are being neglected but for now I’m enjoying that we have a preferential option for Yolŋu and Tiwi people. Compared to the usual preferential option for non-Aboriginal people found in the Royal Darwin Hospital.” -* Dr Sean, journal 20/8/19

Finally, despite the perceived preferential treatment for Yolŋu and Tiwi patients, access to other Aboriginal language interpreters also improved because of the presence of the NT AIS trainer Mandy. During the pilot it was unclear who, amongst health staff, had responsibility to identify patient language needs or book interpreters. Mandy noticed this and took on the role of booking interpreters for the renal team. Dr. Jack appreciated Mandy’s initiative which meant interpreters were often available within an hour. Dr. William said having someone who was responsible to book interpreters embedded in the medical team meant *“family meetings which would have taken a week, were done on the same day.”* It was not possible to track all additional interpreter bookings generated by Mandy however VK observed on just 1 day (26/11/19) Mandy arranged for 3 extra interpreters for patients who spoke Ngaringman, Murrinh-Patha and Ngan’gikurunggurr.

#### Developing knowledge

As outlined above, healthcare provider knowledge of Aboriginal languages spoken in the north of Australia was poor. During period 2, amongst a group of 6 doctors (plus 3 medical students) none knew that Yolŋu Matha referred to a group of dialects which includes Djambarrpuyŋu and Gupapuyngu (VK field notes 25/11/19). Dr. Jack said the lack of knowledge *“speaks to the emphasis that we place on the importance of our Aboriginal patients”*. However, during the pilot, knowledge of dialects and languages spoken in the NT increased amongst doctors with some learning a few phrases. At the bedside of a hospitalised Yolŋu Elder, Dr. William asked Yolŋu Matha interpreter Carly to teach his team the Yolŋu Matha words for ‘good’, ‘no good’ and ‘goodbye’. Interpreters were pleased with this and explained that learning words or phrases showed respect to the patient.

By working closely with interpreters, doctors observed culturally appropriate ways of communicating. For example, in family meetings which included an interpreter, Dr. Jack said he learnt about the importance of listening and remaining silent during interactions to allow patients to consider information. He also learnt that Aboriginal patients make decisions not as individuals but from a collective standpoint considering family, community, culture and medical advice:

“*The presence of an interpreter allowed an understanding of the negotiation processes of health decision making which are so far from our own. We typically view our patients as rational individuals making decisions solely based on the evidence provided without significant influence of a wider range of factors. A dispassionate health consumer, who will always act in self-interest. I think we overestimate our importance and the seemingly irrefutable strength of our recommendations … ..we need to give space and time to our patients and their families to go through processes that I cannot begin to comprehend.” –* Dr Jack, journal 26/11/19

Dr. Sean believed the pilot was a seminal experience for him and others, especially junior doctors and medical students who were still developing their skills. During Period 1, a medical student from the UK said he learnt more from working alongside the Yolŋu Matha and Tiwi interpreter over 10 days than he did from previous cultural awareness courses.

Just as doctors benefited from in situ learning, so too did interpreters. Pre ward round meetings were an opportunity for doctors to explain procedures to interpreters which would then be explained to the patient. VK observed a registrar explaining to interpreter Carly the medical procedure referred to as a “tap”. Dr. Sean said his and Carly’s professional relationship strengthened across 10 days and they developed an efficient communication style. He is confident that with the right support and training all interpreters and doctors can experience the same:

*“She was able to pre-empt things. She's heard me explain this thing ten times, she can actually just crack on. She knows what she's talking about, and she knows what I want to say.* – Dr Sean

#### Challenging attitudes

A mix of attitudes towards communicating with patients in their first language and working with interpreters was exposed. After just 1 day of working with embedded interpreters, Dr. William realised the “*gravity*” of communication: *“I’ve been communicating with people for years who really didn’t understand what we were saying to them.”* With interpreters present*,* Dr. William felt more confident he was delivering culturally competent care. Dr. Sean provided the following example of communicating with and without an interpreter with the same patient:

*“Speaking to a patient in their language allowed us to explain why she’s sick and what we can do for them. They, for maybe the first time, were consented for their procedure in their first language. However, while consented in their first language, doing the procedure at 2pm without an interpreter was very challenging. The requirement to give painful needles to take away the pain of later needles wasn’t something I was able to communicate to this patient in English, their 3rd or 4th language. It was traumatic for everyone involved.”* - Dr Sean, journal 15/8/19

This situation was stressful for the patient and the health providers, so the decision was made to delay the procedure. One week later with the interpreter present the required procedure was completed:

*“Last Thursday, we had a frightened panicked patient, today the use of an interpreter during the procedure allowed me to explain the scans, the needles and what would happen next in the person’s first language. It went well.”* - Dr Sean, journal 22/8/19

Some doctors working on the periphery of the pilot noticed the benefits of working with interpreters and questioned the effectiveness of their own communication. A senior renal registrar started asking her patients if they knew why they were on dialysis. To her surprise, she discovered most patients did not know. She then rectified the situation by booking appropriate interpreters to explain to the patients their condition. Dr. Sean hoped the pilot contributed towards valuing communication in the hospital:

“*The talking bit of medicine - that’s the most important bit of medicine … we have million dollar machines that do fancy scans, most of the diagnoses we make are based on talking to someone”* - Dr Sean

Not all health staff welcomed the pilot experience. During period 1, although the doctor group was enthusiastic, some allied healthcare providers feared embedding interpreters would stymie their capacity to deliver care. Dr. William journaled (14/8/19) MDT members requested a meeting: “*two of the members who called me privately to their offices thought it was unnecessary and was going to undermine their work. I was not sure how and they could not explain how.”* During period 1, doctors and NT AIS staff observed these attitudes and expressed concern some staff appeared to have prioritised themselves over patient needs. Three months later when period 2 commenced, doctors who participated in period 1 had been replaced by a new cohort. On Day 1 of period 2 the new group appeared disinterested in working with interpreters; one team member, who was in favour of the pilot, described the pre ward round MDT meeting, with interpreters present, as a “*shitshow”.* Dr. Jack journaled the same allied health staff who discreetly expressed concern in period 1 now openly displayed contempt: “*Morning handover was rushed, chaotic and very tense, with a degree of hostility between members of the MDT* (multi-disciplinary team) *family.”* After the meeting, doctors divided into two teams to undertake their ward rounds and the Yolŋu Matha interpreter joined one team. Dr. Jack overheard a junior doctor ask the interns:

*“‘Are you coming with us or are you going to join the parade?’ It highlighted the perception among some staff that it is not an integral or even important part of our practice to be able to communicate with our patients. It is viewed as a quaint exercise that has no real impact.”* – Dr Jack, journal 25/11/19

Despite some resistance, after working collaboratively with doctors, the pilot interpreters reported feeling like valued members of the MDT. Period 1 interpreter Carly said: *“We went from strangers, to friends, to family”.* Period 2’s Yolngu interpreter Joanna, who had previously described doctors as intimidating like police, said working alongside Dr. William made her feel valued: *“I felt like I was his shadow”.* Embedded in the medical team with a clearly defined role, Joanna said she felt culturally safe.

*“We were all just one colour. That’s how I felt. I didn’t really see a black or white in the room at all, and there was a lot of different races in there. African, there was a few Asians, non-Indigenous, Yolŋu … It was like we were all the same colour in there.”-* Joanna, Yolŋu Matha interpreter

After working across both periods 1 and 2, NT AIS trainer Mandy confirmed interpreters were “*feeling much more valued and comfortable with medical staff”* but said further work was required to improve relations to ensure sustainable change. Mandy was also concerned the negative attitudes previously felt by Aboriginal interpreters were also experienced by Aboriginal patients. Mandy thought health staff lacked an awareness of patient needs beyond the biomedical and appeared insensitive and unkind to Aboriginal peoples*: “I could just feel body language”.* Mandy was hesitant to label the attitudes as racist, fearing patients may experience a backlash:

*“Racism is a very big word, and maybe it’s their ignorance and not understanding Aboriginal people’s ways … and not taking into account that they’ve got to come from community, leave their country behind and family … to get their treatment. -* Mandy, NT AIS trainer

By participating in the pilot Dr. Jack said he and his colleagues started to talk about patients “*in their own humanity”* which challenged racist stereotypes and changed attitudes:

*“You’re using interpreters and you have an actual meaningful discussion with someone … it gets you to understand who they are, and I think understanding their wishes is mandatory. I think that if we’re seeing patients without actually understanding what they want and whether they consent to something, that’s criminal.”* – Dr Jack

Communicating with patients in their first language builds trust between patient and provider which is required to deliver culturally safe health care. Yolŋu Matha interpreter Carly said without effective communication “*nothing works*”. She continued: “*communication is the life of any relationship”.*

### Post-pilot: opportunities and barriers to sustainable change

Systemic change is required to ensure the positive changes experienced by individuals during the pilot can be experienced more widely. Doctors and interpreters believed the pilot showed how medicine should be delivered in the NT. Reflecting on his experience Dr. Sean declared:

*“English is not the language of the Royal Darwin Hospital … There's many languages that are the language of the Royal Darwin Hospital, and it was quite nice for two weeks to be efficient and be able to be a doctor in a hospital where I don't speak the language.”* – Dr Sean

To ensure the model is sustainable, the following opportunities and barriers need to be considered. Firstly, more cultural education is required. Secondly the lack of trained Aboriginal language interpreters needs to be addressed. Thirdly policies are required to ensure sustainable change.

#### Cultural education

During the pilot Dr. William, wanting to praise the interpreter, said “*I don’t need cultural awareness training, I just need an interpreter.”* (VK field notes 14/9/19) However Mandy explained intercultural communication requires more than an interpreter because “*even when an interpreter’s there, that white person, the English speaker, can say something wrong.”* Incidents were related in which patients were offended by attitude and tone. In one situation, a patient told Mandy that a healthcare provider was “*too pushy”.* Mandy feared staff would resist cultural training which was confirmed by Dr. Jack who journaled (25/11/19) “*the resistance is palpable in eyerolls and groans*”. He explored the idea further in an interview saying that cultural education was seen as *“an imposition that’s in the way of getting on with our business”* but then also suggested TEHS should mandate all staff learn a language indigenous to the NT:

*“Maybe they should just say, ‘Oh, if you haven’t learned an Aboriginal language in your first five years of being here, then we’re not going to renew your contract’*.” – Dr Jack

#### More trained interpreters

A lack of trained interpreters is a barrier to implementing sustainable change. For example, Kunwinkju was the third most spoken language on the renal ward during the pilot however there was only one Kunwinjku interpreter in Darwin employed by the NT AIS and they were working for the justice system. Doctors suggested it may be beneficial to employ interpreters directly at the hospital to ensure access and to build a cohort of health interpreters. Some interpreters felt under-prepared working in the health setting because the NT AIS was unable to deliver consistent health training to interpreters over the last 5 years. NT AIS trainer Mandy was concerned the hospital did not have appropriate systems and cultural knowledge to safely employ and support Aboriginal interpreters directly. Instead, she hoped the two organisations could develop training together to ensure interpreters became familiar with health terminology and familiar with hospital processes. Until more interpreters are trained and employed, Joanna suggested RDH patient lists could be emailed to the NT AIS each afternoon so staff could identify language needs based on patient last names and book interpreters for the following day:

*“It’s just a matter of an email, and boom, boom, boom – Mandy’s really good at picking up someone out of nowhere. Get the list to the bookings team: this is the patients. They can identify the most needed at that time and then send them out.”-* Joanna, Yolŋu Matha interpreter

#### Policies

Finally, policies are required to counter resistance and to ensure changes are not dependant on frontline individuals. Across the pilot, doctors led by the specialist Dr. William were communicating respectfully and effectively with patients but when Dr. William completed his rostered 2 weeks as leader, communication changed. Dr. Sean described another specialist’s style of communication as follows: “*the boss’s style of practicing medicine, is standing at the end of the bed with his arms folded shouting for a few minutes and walking on.”* Dr. Jack believed it will take a “*momentous effort*” to see the model embedded in the hospital and Mandy feared change will only occur after the institution or individuals face penalties:

*“not until something drastic happens and they've got a compensation claim put in, or a coroner's report...It's a lot cheaper to get an interpreter than to go on your merry way and think that everyone understands good English.”-* Mandy, NT AIS trainer

Participating doctors and interpreters would like the model of embedded interpreters in the renal team to continue. They also agreed there is scope to adapt the model for other divisions within the hospital. Dr. Sean proposed an idea that he said would *“fly in the face of medical tradition”.* He suggested that RDH medical teams be arranged to work with language groups which would allow healthcare providers, interpreters, and patients to develop relationships.

*“And surgery would work slightly differently because of the demands of surgery, but I think on a general medicine team, you could... general medicine East Arnhem, general medicine the Daly region … But you have interpreters 8:00 to 4:00, Monday to Friday, who then get to know the doctors, get to know the patients, get to know how the team works”.* – Dr Sean

## Discussion

This paper documents hospital-based healthcare providers and interpreter attitudes towards working together at RDH and the changes which occurred after interpreters were embedded in a renal team over 4 weeks. The analysis reveals benefits and challenges for all involved. Benefits for doctors included improved knowledge of Aboriginal languages and communication styles and increased confidence in working with interpreters. Collaborating consistently with interpreters resulted in doctors feeling more culturally competent when working with Aboriginal language speaking patients. During the pilot, interpreters shifted from feeling unwelcome and undervalued [37] to respected co-healthcare professionals and valuable allies; an approach supported by previous research [48]. Additionally, interpreter’s health literacy improved, and they became active participants in the MDT sharing power and responsibilities with doctors to ensure patient wellbeing. This model of working “with” not “next to” [48] clinicians contrasts with guidelines which present interpreters and healthcare providers as separate. These beneficial outcomes occurred because doctors changed their behaviour which allowed interpreters to surpass the “invisible role as mere linguistic conduits” [48]. Our research found, culturally competent healthcare providers, who collaborate with Aboriginal language interpreters, have the potential to deliver culturally safe care [5]. Aboriginal language speaking patients who feel culturally safe have better health trajectories which can result in less demand on health services [5]. This is referred to as “interest convergence” [49]. Critical race theorists argue when the interests of the “the dominant group, namely White people” converge with those experiencing discrimination, change is more likely to occur [50].

The discussion will now turn to challenges identified by primarily focusing on the attitudes and behaviour of healthcare providers. It is vital to understand the healthcare providers experience because cultural safety places the onus for change on the healthcare provider and the hegemonic institutions [3, 28]. Through understanding healthcare provider perspectives insights are gained into how health systems reproduce inequitable health outcomes [51].

Before the pilot, doctors’ attempts to communicate with patients in their first language were thwarted by perceived hospital priorities. Participating doctors were frustrated and disheartened by their inability to work with Aboriginal language interpreters but attempts to engage interpreters were often impeded by time pressures. Aligning with US research, we found doctors made decisions “about interpreter use by weighing the perceived value of communication in clinical decision making against their own time constraints” [7]. We also found patients who did not converse may be preferred by some providers who aimed for efficient ward rounds. Doctors are taught to control a bedside consult by using a “medical voice” to manage content and duration of the conversation [52]. While important for obtaining required aspects of the medical history, this communication style has been described as “an apparatus of colonisation” used to control Indigenous peoples [53]. During the pilot, doctors changed their communication style to work collaboratively with interpreters thereby testing the conviction that spending time communicating with a patient was inefficient and ineffectual. With interpreters present, the duration of bed side consults extended from 10 min to in some cases 1 hour. Doctors were genuinely listening to patients, which built trust between patient and provider, thereby rehumanising the patient and reducing the power differential [5]. Previous research has asserted investing time communicating with Aboriginal language speaking patients in their first language will have “immense payoffs over the long term.” [54] Our research found after having consistent access to Yolngu Matha and Tiwi interpreters patients felt culturally safe, health trajectories improved and there was a reduction in so called “frequent flyer” patients re-presenting to hospital [5]. As reported here, we also found when doctors invested time in culturally safe communication practices, they were more satisfied with the culturally competent care they were delivering.

Doctors’ attempts to work with interpreters were also stalled by unconscious and overt individual bias. Research suggests that about 75% of Australians have unconscious bias against Aboriginal and Torres Strait Islander peoples [55]. As RDH is a microcosm of broader society, negative perceptions found outside the hospital can be replicated inside the hospital. Furthermore, hospital based health professionals who work long hours in stressful environments where decisions need to be made quickly are more prone to making decisions based on unconscious bias [56]. It is also vital to recognise that medicine has a history of systemic racism [57, 58]. Systemic racism has been defined as the failure of the “system to provide an appropriate and professional service to people because of their colour, culture, or ethnic origin’ [59]. In Australian hospitals historically, Aboriginal patients were segregated and treated in separate wards. At one Top End hospital, the so called “Native Ward” only closed in 1979 [60]. This is in living memory of both long-term health providers and patients. Whilst overt segregation policies no longer exist in Australia, the insidious convention continues to manifest in the colonised nation as described above by doctors. By increasing the number of Aboriginal professionals in the hospital ie. interpreters, the internalized ideologies of non-Indigenous healthcare providers that Aboriginal peoples were deviants, perpetrators or victims was challenged by counter knowledge [47] offered by NT AIS staff. These opportunities assisted in correcting the skewed perception of Aboriginal peoples and lead to some healthcare providers, experiencing what King [47] has referred to as “transformative emotional growth experiences”. Participating doctors who supported this new model of working with interpreters had a level of “critical consciousness” [61, 62] which enabled them to reflect on their own “assumptions, biases, and values” and the institutions in which they work [61]. As a result of their critical consciousness they tested a new way of working with interpreters which required a change in their behaviour to improve health service delivery [55, 63]. Whilst it may be challenging for the anti-racist healthcare provider to accept bias, racialised thinking is virtually inevitable [63, 64] and when accepted, opportunities for change occur as observed in this pilot.

Another challenge which impeded interpreter mediated communication was the hospital culture. RDH staff were socialised into an institution which diminished Aboriginal cultures, as displayed by poor patient language documentation [37], low attendance rates at cultural awareness training [36], low uptake of Aboriginal interpreters [27], and low levels of staff knowledge of Aboriginal languages. Low uptake of Aboriginal interpreters has been blamed on supply issues. However, as we observed even when interpreters were readily available resistance continued. It has been argued this occurs because Aboriginal peoples are expected to assimilate into English speaking Australia [65]. This assertion is supported by evidence which states interpreters of migrant languages are more common than Aboriginal language interpreters in the Australian health care system [24]. Regarding cultural education, before the pilot, approximately 30% of TEHS staff had attended cultural awareness training [36]. Low attendance could imply staff disinterest, but research found TEHS staff wanted more cultural education and in fact low attendance was more likely attributable to the organisational decision to offer cultural education outside of paid work hours [36]. This has since changed [66]. During the pilot, we found further evidence that cultural education is valued by TEHS staff. Cultural education in the form of ‘working with interpreter training’ was delivered as a part of medical training curricula. Initially doctors appeared unconvinced of the value of the training as indicated by the admission it was “squeezed in”. However, after experiencing ‘working with interpreter training’ which included information on Aboriginal languages spoken in the NT, doctors were convinced the training was invaluable, stating it should be mandated. There are two major benefits to incorporating cultural education into the clinical training curricula. Firstly, when training is delivered during the clinician’s workday, it indicates to staff that the organisation values cultural competency as much as clinical competencies [36]. Secondly, attendees can quickly translate learnings into practice thereby testing out and normalising behaviour change [67].

The pilot also identified patterns of ingrained behaviour requiring institutional attention to ensure the delivery of culturally safe care. Firstly, responsibility for booking interpreters should be delegated to identified staff members in each MDT. If patient languages were methodically documented and information provided daily to the NT AIS, the service may be able to prepare casual staff for work the following day. Secondly, we identified two common justifications as to why interpreters were not utilised. Staff assert interpreters are not required because the patient speaks “good English”. The judgment is made based on conversational English not by using a validated assessment tool [5]. Once the assertion is made it is taken as fact, and rarely questioned by colleagues. The habit of judging a patient’s English proficiency must be overturned. It is the language proficiency of the provider that requires assessment [5]. If the provider does not speak the patient’s language, an interpreter is required. This is culturally safe patient centred care. The concept is now promoted amongst TEHS staff, but work is still required to educate staff on the necessary paradigm shift. Considering the cultural and language diversity amongst TEHS staff, about 22% speak English as a second language [68], it could be assumed the value of communicating in first language would be appreciated as indicated by Dr. William. However, healthcare providers appeared to accept the hegemonic Australian culture, the culture of medicine and hospitals over their own understanding of the importance of communicating in first languages. The acceptance of White institutionalised norms, by some healthcare providers, revealed a lack of critical consciousness [62] which has been called dysconscious racism [47]. Dysconsciousness is an uncritical habit of mind that justifies inequity by accepting the status quo [47]. Dysconcious racism risks patient safety [69]. Staff also commonly state patients do not require an interpreter because they did not request one. This assertion ignores that all exchanges between healthcare providers and patients are “power laden” in favour of the provider [3, 4]. This idea was explained by Aboriginal linguist Gloria Brennan in a 1979 Australian government commissioned report on the need for Aboriginal languages interpreters in hospitals: *“It is generally assumed that the more powerful of the two parties will get his message across.*” [70] Healthcare providers control both clinical treatment and communication. Just as a patient is not expected to request a nephrologist or a nurse, they should not be expected to request an interpreter. We acknowledge these justifications may have developed in reaction to a history of unsatisfactory experiences in which interpreters were unavailable. However, these approaches create a self-perpetuating cycle of staff dissatisfaction, and both statements contribute to a culturally unsafe service. The assertions dissociate Aboriginal peoples from their culture and deny Aboriginal peoples the right to speak their language, as deemed a human right by the NT Ombudsmen [71] and set out by the United Nations Declaration on the Rights of Indigenous Peoples [72]. These patterns of behaviour can be addressed through better training as described above and updated hospital policies which could be disseminated to staff through an internal marketing campaign.

As per critical theory, we purposefully focussed our discussion on issues the institution can address as hospitals are regarded as being considerably resistant to change [73]. However, our research also revealed issues requiring attention from the NT AIS. Future models must consider how best to support, develop and retain the Aboriginal interpreter workforce [71]. Regarding support, Aboriginal interpreters often face the same social and cultural determinants of health which lead to their family members being hospitalised as patients. As we saw during the pilot, one interpreter required treatment from the Emergency Department twice during a 5-day work period and another had a family member hospitalised during the pilot study. Employers must understand and adapt to the personal circumstances, family and cultural obligations interpreters juggle alongside the expectations of non-Indigenous colleagues who work within “‘Western’ models of clinical governance and management” [74]. Regarding development, there is a small pool of trained Aboriginal interpreters overall and even fewer trained in health communication. NT AIS interpreters require health training to ensure they are equipped, and confident, to work in the clinical setting. As suggested by Mandy from the NT AIS, this training could be developed as a collaboration between the NT AIS and the NT Department of Health. In terms of retention, the small number of trained interpreters may be associated with employment conditions. All interpreters involved in the pilot were employed casually by the NT AIS. Casual employees face irregular and potentially insufficient work hours, resulting in fluctuations in earnings and are also much less likely than permanent employees to have access to on-the-job training [75].

In the 18 months since this pilot study was undertaken, the hospital has funded employment of up to four part-time interpreters, in addition to contracting interpreters from the NT AIS. It is a positive change which will require sustained education of the hospital staff regarding the delivery of culturally safe care and careful mentoring and support for the interpreters. In consultation with researchers, TEHS has also developed, and adopted, new training modules including the Ask the Specialist podcast [76] which promotes the importance of culturally competent communication with and without Aboriginal interpreters.

A methodological strength of the study was the in-depth qualitative research which revealed dysfunction and the potential for change to redress inadequate systems [77]. We acknowledge this specific model of embedding interpreters in a medical team during morning ward rounds may not be suitable for other hospital departments such as the Emergency Department. However, our findings reveal that barriers to interpreter use stretch beyond the pragmatic issue of interpreter availability and deployment. As suggested, work is required to address the individual and systemic racism which diminishes Aboriginal cultures in health care. We also acknowledge each healthcare provider subgroup lacked gender diversity however this arose from the pragmatic approach which reflected consent processes and staffing at the time.

## Conclusion

This model of Aboriginal interpreter-mediated communication to improve the delivery of culturally competent care provides a viable alternative to the current unsatisfactory approach. Systemic changes are required to ensure the benefits of collaborating with interpreters during the pilot are sustained and scaled up. Continued education of hospital staff about the delivery of culturally safe care, together with mentoring and support for interpreters to ensure a culturally safe workplace should be prioritised. We have provided qualitative evidence regarding the value of culturally competent and interpreter mediated communication in hospital, paving the way for work to examine short term and intermediate cost and health benefits. We contend that investment in culturally safe communication is likely to rival investment in other aspects of healthcare such as expensive diagnostic machines.

## Data Availability

Data from the study are not publicly available due to ethical considerations. Data may be available from the corresponding author on reasonable request.

## Abbreviations

AHP: Aboriginal Health Practitioner
MDT: Multi-Disciplinary Team
NT: Northern Territory
NT AIS: Northern Territory Aboriginal Interpreter Service
PAR: Participatory Action Research
RDH: Royal Darwin Hospital
TEHS: Top End Health Service
